# Case Report: Virtual reality training for phantom limb pain after amputation

**DOI:** 10.3389/fnhum.2023.1246865

**Published:** 2023-12-01

**Authors:** Manabu Yoshimura, Hiroshi Kurumadani, Junya Hirata, Katsutoshi Senoo, Kozo Hanayama, Toru Sunagawa, Kosuke Uchida, Akio Gofuku, Kenji Sato

**Affiliations:** ^1^Faculty of Rehabilitation, Kawasaki University of Medical Welfare, Okayama, Japan; ^2^Graduate School of Biomedical & Health Sciences, Hiroshima University, Hiroshima, Japan; ^3^Department of Rehabilitation Medicine, Kawasaki Medical School, Okayama, Japan; ^4^Quality Assurance Center, Graduate School of Interdisciplinary Science and Engineering in Health Systems, Okayama University, Okayama, Japan; ^5^Department of Anesthesiology & ICM, Kawasaki Medical School, Okayama, Japan

**Keywords:** virtual reality, phantom limb pain, amputation, upper limb activity, rehabilitation, motor imagery

## Abstract

Several reports have demonstrated the effectiveness of neurorehabilitation, such as mirror therapy or virtual reality, in treating phantom limb pain (PLP). This case study describes the effect of virtual reality training (VRT) on severe, long-term PLP and upper limb activity on the amputated side in a patient who underwent digit amputation 9 years prior. A woman in her 40 s underwent amputation of 2–5 fingers 9 years prior due to a workplace accident. She experienced persistent pain in the palms of her hand near the amputation sites. A single case design (ABA’B’) was applied. Periods A and A’ were set as periods without VRT intervention, and Periods B and B’ were set as periods with VRT intervention. Periods A, B, A’, and B’ lasted 4, 10, 8, and 10 weeks, respectively. VRT was a task during which visual stimulation and upper limb movements were linked. The task consisted of catching a rolling ball in the display with a virtual hand, operated with both hands using a controller. VRT was performed once every 2–4 weeks for 30 min. Pain intensity was assessed using the short-form McGill Pain Questionnaire-2. Bilateral upper limb activity was measured continuously for 24 h using a triaxial accelerometer attached to the right and left wrist joints. The pain intensity was 147/220 points during Period A, 128 points during Period B, 93 points during Period A’, and 100 points during Period B’, showing a gradual decrease. Upper limb activity occurred mainly on the intact side during Periods A and B, whereas the activity on the amputated side increased 2-fold after Period A’, and both upper extremities were used equally. Virtual reality training resulted in reduced pain intensity and increased activity in the upper limb. VRT may have induced reintegration of the sensory-motor loop, leading to a decrease in the PLP intensity. The upper limb activity on the amputated side may have also increased with the pain reduction. These results suggest that VRT may be valuable in reducing severe, long-term PLP.

## 1 Introduction

Phantom limb pain (PLP) occurs in the missing part of a limb after amputation, and is an intractable pain that tends to become severe and chronic (Sherman et al., 1984; Jensen et al., 1985). Approximately 48% of the patients who undergo amputations experience PLP more than once a day, and 64% experience moderate-to-severe PLP (Kooijman et al., 2000). Although the mechanism of PLP remains unclear, abnormal impulses from neuromas, neurons in the spinal cord, and the excitability of the central nervous system appear involved (Collins et al., 2018). There is consensus that disturbance of sensory-motor loops is involved in generating PLP. Therefore, various practical approaches, such as mirrors, virtual reality (VR), and hand-mental rotation tasks, have focused on reintegrating sensory-motor loops (Ramachandran et al., 1995; Nico et al., 2004; Sano et al., 2016; Osumi et al., 2019; Yoshimura et al., 2022).

Several previous studies have shown mirror therapy’s effectiveness in treating PLP (Ramachandran et al., 1995; Chan et al., 2007; Barbin et al., 2016); however, the monotonous nature of movements makes compliance cumbersome. Therefore, this study focused on VR because of its immersive nature, which can be described as a feeling of being in a VR space, and motivational effects. VR simulates an environment where user experiences are comparable to the real world (Weiss and Jessel, 1998). Previous studies have shown that VR training (VRT) promotes the recovery of upper limb function in patients with stroke or Parkinson’s disease (Turolla et al., 2013; Kiper et al., 2018; Cikajlo and Potisk, 2019). Marcos et al. reported that VRT is a valuable technology that promotes better movement and cognition (Perez-Marcos et al., 2018). Our previous study also reported that VR enhances the immersive experience during action observation compared with tablet devices (Yoshimura et al., 2020).

Previous studies have reported that VRT induced the illusion of two-handed manipulation by projecting a mirror image of an intact hand in a virtual space, thereby decreasing PLP (Osumi et al., 2019). Although mirror images were not used in the current VRT, the intact and amputated hands could be moved separately. Furthermore, we hypothesized that the vibratory stimulus to the controller generated when the virtual hand grasps the ball induces a motor illusion and reduces PLP intensity.

Previous PLP intervention studies focused only on pain intensity. This study is the first to measure changes in amputated and non-amputated limb activity in daily life associated with changes in pain intensity. This study aimed to examine the changes in PLP intensity and upper limb activity using VRT performed in a single-case design in a patient with PLP after finger amputation.

## 2 Case presentation

**FIGURE 1:**
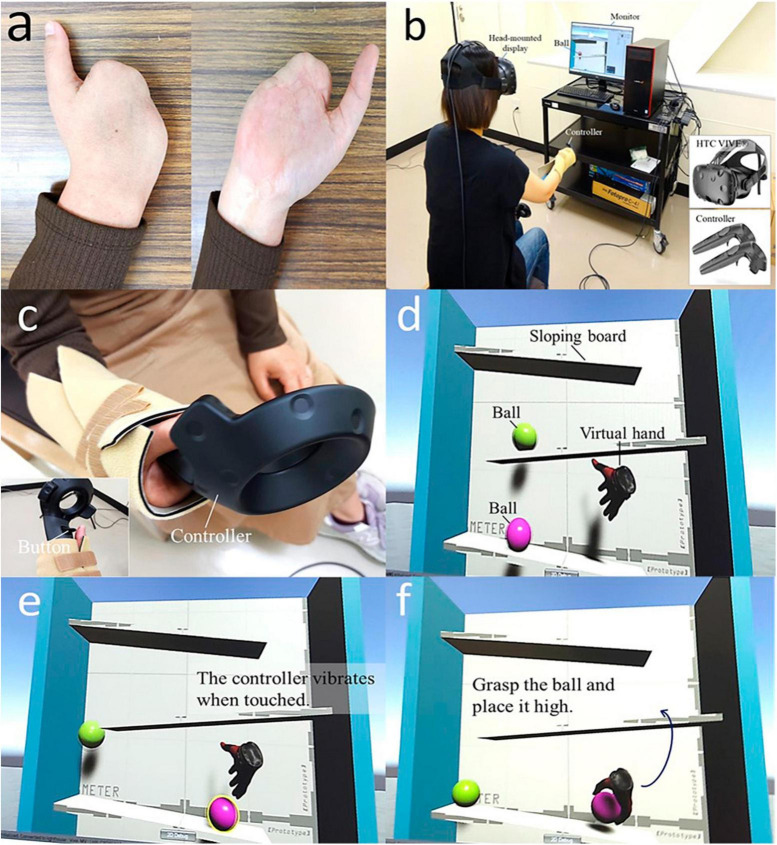
**(a)** Dorsal and palm on the amputated side. **(b)** Scene of VRT implementation with head-mounted display. **(c)** Controller fixed to the stump on the amputated side. **(d–f)** A ball rolling on a board in the virtual space is grasped by a virtual hand and placed high on the board.

A 42-year-old woman underwent amputation of 2–5 fingers following a workplace accident 9 years previously (Figure 1a). The pain due to the amputation of the fingers penetrated the palm. The daily maximum pain intensity was 100/100 mm, with an average 82/100 mm score on the visual analog scale (VAS). The patient had to quit her job after the injury. However, she could not use the affected limb daily due to severe pain and was often confined to the bed during the day. Mirror therapy for PLP was performed at another hospital, but she had a strong aversion and discomfort while looking in the mirror, which made it difficult to continue the therapy. Although various other interventions for pain, such as alternating baths, were performed, no improvement in PLP was observed. Two excisional surgeries were performed, including removing a palmar nerve seed.

The patient received tramadol hydrochloride, neurotropin tablets, duloxetine hydrochloride, suvorexant, etizolam, and brotizolam for the management of pain, and the amount of drug and frequency of rehabilitation remained constant during all periods. This study was approved by the Ethics Committee (5466-00); written explanations were given to the patient, and her consent was obtained.

### 2.1 Intervention

Virtual reality training was performed using a head-mounted display in the chair position, and a controller was used to control the virtual hand (Figure 1b). The head-mounted display was an HTC VIVE, and the controller was fixed to a stump for operation (Figure 1c).

The task was created in our research team using Unity and involved repeatedly grasping a ball rolling on a sloping board in a virtual space with a virtual hand (Figures 1d–f). The patient repeatedly grasped the ball with the virtual hand and placed it at a higher position on the sloping board. The virtual hand was opened and closed by pressing a button at the bottom of the controller with the thumb, and a vibration stimulus was input to the controller when the ball was touched. The number of balls and the speed at which they rolled could be adjusted based on the execution of the VRT. The images seen by the patient during the VRT could be viewed on the monitor in front of her.

After VRT, feedback was provided by the occupational therapist on the changes in the range of motion and movement speed of the shoulder joint using videos taken during the VRT sessions. Activities that could be performed daily were discussed, and goals were shared.

Virtual reality training was performed every 2–4 weeks for 30 min. In this study, a single-case design (ABA’B’ type) was used to examine the effectiveness of VRT. Single-case design is a case study method that systematically manipulates the treatment, systematically measures and evaluates the performance, and has the experimental element of capturing new intervention methods in a preliminary research manner (Barnett et al., 2012). The ABA’B’ design used in this study is a basic single-case design for examining interventions and sustained effects.

**FIGURE 2:**
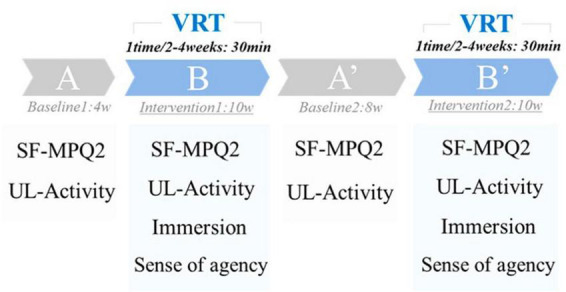
Protocol.

Period A was the first baseline period (without VRT, 4 weeks), Period B was the first intervention period (with VRT, 10 weeks), Period A’ was the second baseline period (without VRT, 8 weeks), and Period B’ was the second intervention period (with VRT, 10 weeks) (Figure 2).

### 2.2 Measurements

Pain intensity was assessed using the short-form McGill Pain Questionnaire-2 (SF-MPQ2). SF-MPQ2 is a questionnaire that assesses the pain intensity on a scale of 0 (none) to 10 (worst possible pain) for 22 types of pain and has been used in many reports (Dworkin et al., 2009; Maruo et al., 2014). This assessment results in a 220-point scale, with higher scores indicating higher pain intensity. The SF-MPQ2 was administered during the last week of each period and was used as the score for each period (Figure 2).

Bilateral upper limb activity was assessed using Actigraph’s GT9X for 24 consecutive hours. GT9X was applied to the right and left wrist joints, and upper limb activity was measured for 24 consecutive hours, excluding bathing. In accordance with previous studies, the duration of each application was 24 h (Bailey et al., 2015; Hayward et al., 2015). The sampling rate was set to 30 Hz. Vector magnitudes (VM) combined by the square root of the sum of squares [√(X^2^ + Y^2^ + Z^2^)] of each axis (X, Y, and Z-axes) were downloaded from the dedicated software ActiLife6 and used for analysis. Upper limb activity was assessed during the last week of each period and used as the score for each period. The same day of the week was used for the measurement, and the patient was asked to record her activities on paper to confirm that no special events occurred on the day of the assessment (Figure 2).

A visual analog scale assessed immersion during the VRT, with scores ranging from 0 (not immersive) to 100 (immersive). The virtual hand’s sense of agency (“I caused this movement”) was rated on a 7-point Likert scale from 1 to 7, with 1 indicating the lowest sense of agency and 7 indicating the highest. The sense of immersion and agency was measured at each intervention during Periods B and B’, and the average value was used as the score (Figure 2).

## 3 Results

**FIGURE 3:**
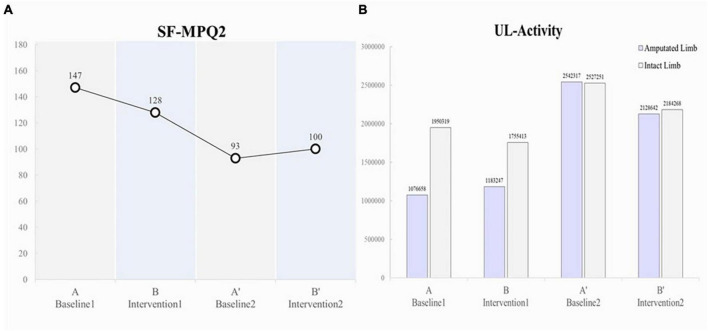
**(A)** Changes in SF-MPQ2. **(B)** Changes in upper limb activity.

The pain intensity (SFMPQ-2) decreased gradually, from 147/220 during Period A to 128/220 during Period B, 93/220 during Period A’, and 100/220 during Period B’ (Figure 3A). The intensity of gnawing pain, tiring-exhausting pain, punishing-cruel pain, and piercing pain decreased (Table 1). In contrast, the pain intensity decreased during the period A, but the pain that was enhanced during the period B’ was throbbing, stabbing, and sharp. After period B’, the pain intensity was maintained at 100–105/220, lower than the initial score.

**TABLE 1:** Subcategories of SF-MPQ2.

Subcategories	A	B	A’	B’
**1. Throbbing pain**	**6**	**6**	**3**	**6**
2. Shooting pain	8	8	5	5
**3. Stabbing pain**	**6**	**6**	**3**	**8**
**4. Sharp pain**	**6**	**6**	**3**	**8**
5. Cramping pain	8	8	9	8
**6. Gnawing pain**	**8**	**8**	**0**	**0**
7. Hot-burning pain	9	9	9	9
8. Aching pain	8	8	8	5
9. Heavy pain	8	8	5	5
10. Tender	0	0	0	0
11. Splitting pain	5	5	5	5
**12. Tiring-exhausting**	**8**	**1**	**0**	**0**
13. Sickening	8	9	9	9
14. Fearful	0	0	0	0
**15. Punishing-cruel**	**8**	**8**	**0**	**0**
16. Electric-shock pain	8	8	3	3
17. Cold-freezing pain	8	3	3	3
**18. Piercing**	**8**	**0**	**0**	**0**
19. Pain caused by light touch	8	8	6	3
20. Itching	3	3	5	5
21. Tingling or “pins and needles”	8	8	9	9
22. Numbness	8	8	8	9
Score	147	128	93	100

Red indicates pain that has decreased in intensity. Blue indicates pain that has decreased in intensity and then increased.

In terms of upper limb activity, the patient pre-dominantly used the intact limb side during Periods A (amputated limb, 1076658; intact limb, 1950319) and B (amputated limb, 1183247; intact limb, 1755413); however, during Period A’, increased activity was observed on the amputated limb side (double that of Periods A and B; amputated limb, 2542317; intact limb, 2527251), and the left and right sides were used equally (Figure 3B).

The immersion score was as high as 95.2 ± 9.6 during Period B and 100 ± 0.0 during Period B’. The sense of agency was similarly high, at 6.4 ± 0.8 during Period B and 7.0 ± 0.0 during Period B’.

During Period A, the patient tended to be confined to bed during the day and used the amputated limb to a lesser degree. The patient also used a Lofstrand cane due to severe pain. During Period B, the patient started VRT and felt the illusion of moving her amputated finger in the VR space due to the early intervention. The patient said, “It is like my missing finger is really moving.” VRT was repeated, and the penetrating sensation of the phantom limb was reduced during the second half of Period B. The patient could also observe her movements objectively while receiving feedback after VRT, as she could not see her movements due to using the head-mounted display during VRT. The patient said, “I did not think I could move like this.” During the middle of Period B, the patient discussed with the occupational therapist that the goal was to use the amputated limb for daily washing, drying, and cooking.

During Period A’, the patient could pick up clothespins with her thumb when drying clothes and hold food with the amputated limb when cooking. The amount of time spent confined to bed during the day decreased, and the amount of activity increased. The patient no longer needed a Lofstrand cane when walking.

During Period B’, the patient said, “I still have pain, but I am able to use the amputated limb naturally in my daily life.” The patient can now wash, cook, and write by picking up the pen with the side of her thumb. In addition, the patient could carry her grandchild, who was born during Period B’, and change her diaper.

## 4 Discussion and conclusion

This study demonstrated that VRT reduced pain intensity and improved upper limb activity in a patient with PLP after finger amputation. The effect of VRT in the present study may have involved the sensory-motor loop. McCabe et al. demonstrated the mechanism of pain appearance due to disruption of the sensory-motor loop (McCabe et al., 2005). In their study, healthy participants held a mirror near the midline of the body, and abnormal sensations, such as pain, numbness, strangeness, and disgust, were elicited in the hand under conditions in which the movement of the left hand in the mirror and the actual right hand behind the mirror were discordant. The study concluded that dissociating visual and somatosensory perception disrupts the sensorimotor loop, eliciting morbid pain and abnormal sensations. In this case, the mechanism of PLP occurrence was considered the loss of feedback between finger movement and sensory information due to amputation of the finger. This resulted in a mismatch between the somatosensory and visual information, which disrupts the sensory-motor loop and causes pathological pain, such as PLP.

Pain reduction by VRT was suggested due to the reintegration of the sensory-motor loop, which induces the illusion of finger movement in the virtual space, resulting in a match between visual and somatosensory information. The use of VRT for the treatment of PLP promotes a sense of ownership (“this is my hand”) and agency (“I caused this movement”) in a highly immersive virtual space (Gallagher, 2000). As a result, motor imagery of the amputated limb is improved, and PLP intensity is reduced. Cole et al. reported that PLP enhances agency and ownership and leads to cognitive recovery of the limb (Cole et al., 2009). Furthermore, visual information is considered the most important sensory information for reintegrating the sensory-motor loop. The hand can be perceived as part of one’s body, and the integrity of the sensory-motor loop can be reconstructed using visual illusions (Cole et al., 2009).

In addition to visual illusions in this study, vibratory stimulation to the controller may have been one of the factors that encouraged the induction of motor illusions. Sano et al. performed VRT on patients who had undergone amputation and patients with brachial plexus palsy (Sano et al., 2016). They reported a higher sense of agency and lesser pain in the condition with vibratory stimulation compared with the condition without vibration stimulation. In the present study, the vibration stimulation of the virtual hand touching the ball also produced a high sense of immersion and agency, suggesting that it may have contributed to the reintegration of the sensory-motor loop.

Virtual reality training showed a reduction in PLP intensity during Period B, and the reduction effect may have persisted during the subsequent Period A’ (Figure 3A). This result suggests that VRT continued to reduce PLP intensity during the intervention and baseline periods. Increased upper limb activity in daily life was also observed with pain reduction. Subsequently, Pain intensity was slightly enhanced in period B’ compared to period A’. This result suggests that the sustained effects were attenuated in Period B’. Table 1 shows that the pain enhanced in period B’ was somatosensory-related pain, such as “throbbing pain,” “stabbing pain” and “sharp pain.” It has been reported that somatosensory-related pain is more difficult to reduce than PLP-associated kinesthesia-related pain, such as “gnawing pain” and “piercing pain” (Osumi et al., 2019). The present report suggests that although somatosensory-related pain showed temporary reduction at period A’, it was difficult to sustain the pain reduction effect of VR, and pain intensity increased at period B’.

Before VRT, the patient had reduced the use of the amputated side daily because of persistent pain. Thus, the limb may have been in a state of learned non-use (Taub, 1980) for 9 years after the disease onset. However, she could move the virtual hand and felt the illusion of movement. Furthermore, through video feedback of her movements during the VRT and shared goals with the occupational therapist, she noticed improvements in the range of motion and speed of the amputated limb she was unaware of. Consequently, she could relearn how to use the amputated limb in daily activities, leading to increased activity. In particular, the increase in bilateral upper limb activity from B to A’ was due to increased activity in bimanual activities, such as drying clothes and cooking, and increased activity was observed in the intact and amputated limbs. The results of this study were based on a single case and may differ from studies based on multiple cases. Therefore, further studies with more cases are needed. In addition, VRT was performed every 2–4 weeks for 30 min. The frequency and duration of the necessary intervention were not clarified in this study and need to be verified to clarify them in the future.

In the future, measures should be taken to implement the system for finger and higher-level amputations, such as forearm and upper arm amputations. Since it is difficult to open and close the virtual hand using the buttons on the controller for high-level amputations, we plan to use a foot switch on the same side of the foot to operate the hand.

The use of VRT resulted in a reduction in pain intensity and an increase in upper limb activity in a patient with long-term PLP after finger amputation. Thus, VRT may be helpful for refractory pain in the upper extremities.

## Data availability statement

The raw data supporting the conclusions of this article will be made available by the authors, without undue reservation.

## Ethics statement

The studies involving humans were approved by the Ethical Review Board of Kawasaki Medical School. The studies were conducted in accordance with the local legislation and institutional requirements. The participants provided their written informed consent to participate in this study. Written informed consent was obtained from the individual(s) for the publication of any potentially identifiable images or data included in this article.

## Author contributions

MY, HK, JH, KS, KH, TS, KU, AG, and KS defined the study protocol. KU, AG, and KS developed the VR system. KS was responsible for participant recruitment. MY, KU, AG, and KS conducted the experiments. MY and JH analyzed and interpreted the data and wrote the manuscript. HK, JH, KS, KH, TS, KU, AG, and KS provided advice on writing the manuscript. All authors contributed to the article and approved the submitted version.
